# Development and Long-Term Acceptability of ExPRESS, a Mobile Phone App to Monitor Basic Symptoms and Early Signs of Psychosis Relapse

**DOI:** 10.2196/11568

**Published:** 2019-03-29

**Authors:** Emily Eisner, Richard James Drake, Natalie Berry, Christine Barrowclough, Richard Emsley, Matthew Machin, Sandra Bucci

**Affiliations:** 1 Division of Psychology and Mental Health, School of Health Sciences Manchester Academic Health Science Centre University of Manchester Manchester United Kingdom; 2 Greater Manchester Mental Health National Health Service Foundation Trust Manchester United Kingdom; 3 Department of Biostatistics and Health Informatics King's College London Institute of Psychiatry, Psychology and Neuroscience De Crespigny Park London United Kingdom; 4 Division of Informatics, Imaging & Data Sciences Manchester Academic Health Science Centre University of Manchester Manchester United Kingdom

**Keywords:** schizophrenia, psychotic disorders, recurrence, telemedicine, mobile health, mHealth, eHealth, mental health

## Abstract

**Background:**

Schizophrenia relapses are common, have profound, adverse consequences for patients and are costly to health services. Early signs interventions aim to use warning signs of deterioration to prevent full relapse. Such interventions show promise but could be further developed. This study addresses 2 developments: adding basic symptoms to checklists of conventional early signs and using a mobile phone app ExPRESS to aid early signs monitoring.

**Objective:**

This study aimed to (1) design a pool of self-report items assessing basic symptoms (Basic Symptoms Checklist, BSC); (2) develop and beta test a mobile phone app (ExPRESS) for monitoring early signs, basic symptoms, and psychotic symptoms; and (3) evaluate the long-term acceptability of ExPRESS via qualitative feedback from participants in a 6-month feasibility study.

**Methods:**

The BSC items and ExPRESS were developed and then adjusted following feedback from beta testers (n=5) with a schizophrenia diagnosis. Individuals (n=18) experiencing a relapse of schizophrenia within the past year were asked to use ExPRESS for 6 months to answer weekly questions about experiences of early signs, basic symptoms, and psychotic symptoms. At the end of follow-up, face-to-face qualitative interviews (n=16; 2 were uncontactable) explored experiences of using ExPRESS. The topic guide sought participants’ views on the following *a priori* themes regarding app acceptability: item content, layout, and wording; app appearance; length and frequency of assessments; worries about app use; how app use fitted with participants’ routines; and the app’s extra features. Interview transcripts were analyzed using the framework method, which allows examination of both *a priori* and *a posteriori* themes, enabling unanticipated aspects of app use experiences to be explored.

**Results:**

Participants’ mean age was 38 years (range 22-57 years). Responses to *a priori* topics indicated that long-term use of ExPRESS was acceptable; small changes for future versions of ExPRESS were suggested. *A posteriori* themes gave further insight into individuals’ experiences of using ExPRESS. Some reported finding it more accessible than visits from a clinician, as assessments were more frequent, more anonymous, and did not require the individual to explain their feelings in their own words. Nevertheless, barriers to app use (eg, unfamiliarity with smartphones) were also reported. Despite ExPRESS containing no overtly therapeutic components, some participants found that answering the weekly questions prompted self-reflection, which had therapeutic value for them.

**Conclusions:**

This study suggests that apps are acceptable for long-term symptom monitoring by individuals with a schizophrenia diagnosis across a wide age range. If the potential benefits are understood, patients are generally willing and motivated to use a weekly symptom-monitoring app; most participants in this study were prepared to do so for more than 6 months.

**Trial Registration:**

ClinicalTrials.gov NCT03558529; https://clinicaltrials.gov/ct2/show/NCT03558529 (Archived by WebCite at http://www.webcitation.org/70qvtRmZY).

## Introduction

A total of 80% of those with first episode psychosis relapse within 5 years [1,2], which often leads to unplanned admissions [3-5], increased personal distress [6], vocational disruption [7], worse residual symptoms [8], and risk of suicide [9-11]. Interventions using early signs of deterioration (eg, increased anxiety and insomnia) to prompt timely preventative action can forestall relapse [12-14] but could be further developed by improving relapse prediction and increasing engagement with long-term early signs monitoring [15]. This study addresses 2 such developments: (1) adding basic symptoms to checklists of conventional early signs and (2) using mobile phone technology to aid early signs monitoring.

Basic symptoms are subtle, subjective changes in individuals’ experiences of themselves (eg, difficulty managing attention) and the world around them (eg, more vivid colors) that predict first episodes of psychosis [16,17] and might predict relapses [18-20]. The most comprehensive studies to date have been retrospective [18,19]. One prospective study [21] examined 10 items resembling basic symptoms, but the authors did not explicitly state that these were basic symptoms or specify how they were measured [19]. There is a clear need for a well-powered, prospective study to establish whether combining basic symptoms with conventional early signs improves relapse prediction. We plan to carry out such a study using a phone app to facilitate monitoring, in line with recent developments in mobile health (mHealth) for psychosis.

In this study, we describe the development and long-term acceptability of ExPRESS, a phone app that monitors basic symptoms and conventional early signs of psychosis relapse on a weekly basis, and the basic symptoms checklist (BSC), the pool of self-report items used to assess basic symptoms in the app. Both as a research tool and in clinical practice, an app potentially has numerous advantages over face-to-face [22], postal [23], or text message-based [24] assessments of early signs of relapse. An app can be accessed at times and places convenient to the patient, as mobile phones tend to be carried around from place to place and their use is often integrated within daily life [25]. Monitoring early signs with an app is less resource intensive, intrusive, time consuming, and burdensome than weekly visits from a researcher or clinician. People with a schizophrenia diagnosis find apps acceptable for short-term self-monitoring [26] and prefer them to text message–based systems [27]. Native phone apps are preferable to Web-based systems, being less dependent on a good data or Wi-Fi connection and, therefore, more accessible in rural locations and for low-income users [25]. Furthermore, apps can include automated features such as reminders, generation of graphs, and secure upload of data, which might enhance user experience and increase engagement.

We know of 9 published studies that have prospectively assessed symptom course using a phone app [26-34] but none testing an app to monitor early signs of psychosis relapse. Only 1 study to date has evaluated a symptom monitoring app for longer than 6 months [34], and none in a sample with established psychosis. Much current literature relies on satisfaction ratings of apps for psychosis [25,33-35]. Only a few studies include qualitative feedback from people with psychosis regarding actual [28,36,37] or hypothetical [38-41] acceptability of apps to monitor or ameliorate psychosis symptoms. Most had relatively young samples, likely to be more *au fait* with this technology (*digital natives*) than is typical of those with chronic psychosis, and none examined psychosis patients’ experiences of long-term native phone app use.

Integrating user feedback into the design of mobile phone apps and psychological interventions is best practice [28,36,42-44] and improves engagement with digital tools for psychosis [45]. Ben-Zeev et al [25] recommend that researchers publish descriptions of app development, including specific ways the design was influenced by user feedback. Accordingly, we provide details of the ExPRESS app design and how it was changed in response to feedback during beta testing. We then describe a qualitative analysis of in-depth feedback from longitudinal feasibility study participants regarding the actual acceptability of the app (*a priori* themes), as recommended in a recent systematic review [46]. Framework analysis [47] allowed us to also consider *a posteriori* themes to further understand patients’ experiences of long-term symptom monitoring with a phone app. The rich qualitative data provided valuable information on how people with psychosis use symptom monitoring apps, whether they perceive any value from doing so and what challenges they might encounter.

**Figure 1:**
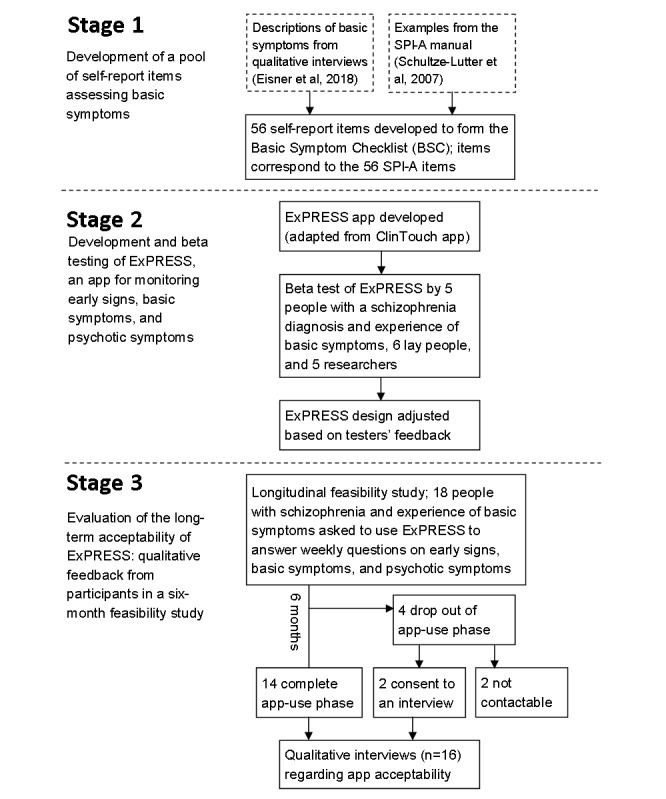
Flow diagram of study design. BSC: Basic Symptom Checklist; SPI-A: Schizophrenia Proneness Instrument, Adult Version.

We describe 3 stages of the study (Figure 1), with the following 3 aims:

1. Stage 1: To design a pool of self-report items assessing basic symptoms (BSC);
2. Stage 2: To develop and beta test a mobile phone app ExPRESS for monitoring early signs, basic symptoms, and psychotic symptoms; and
3. Stage 3: To evaluate the long-term acceptability of ExPRESS by gathering qualitative feedback from participants in a 6-month feasibility study.

## Methods

### Stage 1: Basic Symptom Item Pool

A pool of 56 self-report basic symptoms items was developed to form the BSC. Item wording was based on qualitative interviews in which participants described basic symptom experiences before psychosis relapse [19] and descriptions and examples given in the Schizophrenia Proneness Instrument, Adult Version manual (SPI-A) [48]. Our retrospective study [19] is one of the few to collect quotations from English-speaking participants regarding basic symptoms, making it an ideal foundation for an English-language self-report measure. Items were specifically designed for mobile phone app completion and had a 7-point visual analog scale with 2 anchors (*Not at all*; *A great deal*).

### Stage 2: ExPRESS App Development

#### Overview of ExPRESS

The software was adapted from ClinTouch [26], a symptom-monitoring app assessing 12 Positive and Negative Syndrome Scale (PANSS) [49] and 2 Calgary Depression Scale (CDS) [50] items. ClinTouch and ExPRESS are compared in Table 1. They share their look, feel, and general functionality, but features such as item content and alert frequency differ, as does the period of monitoring studied.

**Figure 2:**
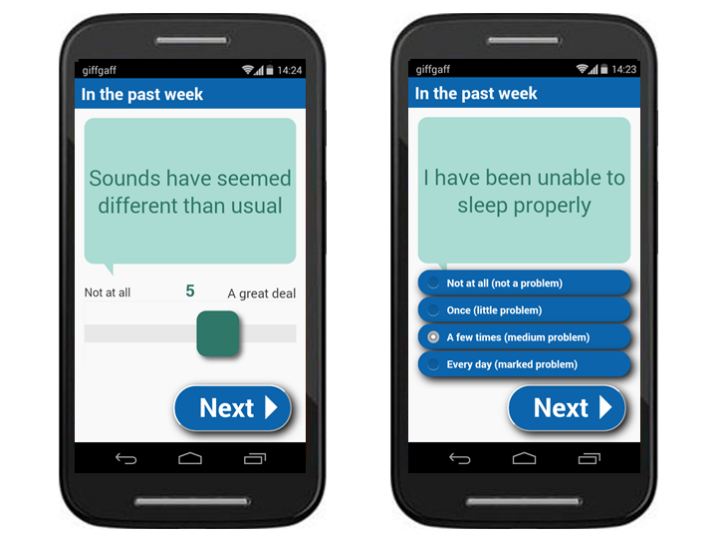
Screenshots of an example basic symptom item (left) and early signs item (right) displayed on the ExPRESS (Experiences of Psychosis Relapse: Early Subjective Signs) app.

ExPRESS includes a pool of 5 PANSS and 2 CDS items, plus items from the BSC, the early signs scale (ESS) [51], fear of recurrence scale (FoRSe) [52], and optional personalizable items. All assessments relate to the past week, with PANSS, CDS, BSC, and personalizable items answered by moving a sliding bar along a 7-point visual analog scale and ESS and FoRSe items scored on a 4-point ordinal scale (Figure 2). Individuals do not use all items but select a subset of relevant items (≤5 from BSC; ≤5 from ESS or FoRSe; and ≤5 personalizable items) for monitoring, together with all 7 PANSS and CDS items. PANSS and CDS items include additional follow-up questions (eg, *This stopped me from doing things*) contingent on the user’s answer to an initial question (eg, *I have heard voices*). The total number of questions to be answered per week ranges from 26 to 53, with the average number asked in this study being 42.

**Table 1:** Comparison of ClinTouch and ExPRESS app design.

App feature		ClinTouch app	ExPRESS app
**Items**			
	Items scored on a 7-point visual analog scale, using a sliding bar with 2 anchors	12 PANSS^a^ items: delusions, hallucinations, suspiciousness, grandiosity, anxiety, depression, guilt, somatic concern, passive apathetic social withdrawal, hostility, excitement, and conceptual disorganization; and 2 Calgary Depression Scale items: depression and hopelessness	5 PANSS items: delusions, hallucinations, suspiciousness, grandiosity, and anxiety; 2 Calgary Depression Scale items: depression and hopelessness; Basic Symptoms Checklist (56 items); and optional personalizable items
	Items scored on a 4-point Likert scale	None	Early Signs Scale (34 items) and Fear of Recurrence Scale (3 items)
	Items that can be personalized (eg, based on baseline interview)	PANSS delusions items (≤2)	Basic Symptoms Checklist (≤5), Early Signs Scale or Fear of Recurrence Scale (≤5), personalizable items (≤5), and PANSS delusions items (≤2)
**Extra features**			
	Wallpaper	Yes	Yes, with new photos
	Daily diary	Yes	Yes
	Graphs of delusions and hallucinations	Yes	Yes
	Useful numbers	Yes	Yes
**Alerts**			
	Frequency of alerts	6 pseudo-random occasions per day	Once per week
	Alert day(s)	Monday, Tuesday, Wednesday, Thursday, Friday, Saturday, and Sunday	Wednesday, 1.30 pm
	Time window for answering the questions	15 min	24 hours
	Snooze options	Snooze for 5 min	First snooze for half an hour and second snooze for 22.5 hours
**Data upload**			
	Data upload	Wireless upload using mobile internet	Wireless upload using mobile internet
	Data storage	Secure server at the University of Manchester	Secure server at the University of Manchester

^a^PANSS: Positive and Negative Syndrome Scale.

ExPRESS alerts the participant to answer questions once a week (1.30 pm on a Wednesday), using a beep and visual notification. The participant has 24 hours to answer. They have the option to snooze the app and receive additional alerts after 0.5 and 22.5 hours. Using the mobile phone’s internet connection, ExPRESS automatically uploads participants’ responses to a secure server maintained by the University of Manchester, where responses are accessible to the research team via a password-protected Web interface.

As in ClinTouch, participants could choose to personalize the app by changing the wallpaper and could view automatically generated graphs of recent responses to delusions, hallucinations, and mood items. There was also a daily diary for the individual’s own reference (not uploaded) and a section containing useful numbers (eg, Samaritans).

#### Beta Testing

ExPRESS was beta tested on a variety of mobile phone handsets by psychology researchers (n=5) and lay people (n=6) for 1 week each. Separate, one-off beta tests were then conducted by patients (n=5) with a schizophrenia-spectrum diagnosis (Diagnostic and Statistical Manual of Mental Disorders, 4th Edition; DSM-IV) and experience of basic symptoms. Participants were given an overview of ExPRESS and were asked to perform a series of in-app tasks (observed by the researcher) and to provide comments using a structured app-evaluation form (Multimedia Appendix 1). Their feedback and the researcher’s observations were collated, and proposed changes to ExPRESS were discussed by the research team. Given the study’s finite resources, namely, computer programmer time, changes that improved the app’s functionality and were in line with its overall purpose were prioritized (eg, adding *in the past week* to all questions to clarify which period they refer to).

### Stage 3: 6-Month Feasibility Study and Qualitative Interviews

#### Feasibility Study

Patients (n=18; convenience sample) were recruited from 3 NHS Mental Health Trusts in the North West of England to participate in a 6-month, single-arm, open longitudinal feasibility study (see study protocol for full details [53]; Research Ethics Committee reference 14/NW/1471). They were trained on ExPRESS (on their own phone, if compatible, or on a study phone) and asked to use it weekly for 6 months or until relapse, whichever was sooner. Participants received supportive telephone calls from the researcher (weekly for the first 4 weeks and monthly thereafter) to encourage participation and troubleshoot any difficulties with app use. Those using ExPRESS on a study phone received weekly text messages to their own phone number to remind them to use the app. For participants giving additional consent, app assessments were sent to a named clinician. Notes on all telephone calls and face-to-face meetings with participants during the follow-up period were included in the study feasibility diary. At the end of follow-up, all participants were invited (via telephone) to take part in a qualitative interview, including those who had dropped out of the app use phase (n=4, of whom 2 were uncontactable). Quantitative findings from the longitudinal feasibility study are reported elsewhere.

Inclusion criteria for the longitudinal feasibility study were as follows: schizophrenia-spectrum diagnosis (DSM-IV); at least 1 acute psychotic episode in the past year (exacerbation of psychotic symptoms lasting at least 2 weeks and requiring a management change, including admission) or at least 2 acute episodes in the past 2 years, including index episode; reporting basic symptoms (assessed using SPI-A), which began or increased before a previous psychotic episode; currently prescribed antipsychotic medication; age over 18 years; fluent in English; fixed abode; sufficiently stable to take part; no current alcohol or drug dependence (Structured Clinical Interview for DSM-IV, [54]); and providing informed consent.

#### Qualitative Interviews

Qualitative interviews were conducted at the end of the 6-month feasibility study by 2 researchers (EE and NB) using a topic guide (Multimedia Appendix 2; piloted with 1 lay participant) covering: item content, layout, and wording; app look and feel; length and frequency of assessments; worries about app use; how app use fitted with participants’ routines; the app’s extra features; and other experiences of using the app. Both interviewers were female PhD researchers with Master’s degrees and experience conducting qualitative interviews; EE had met all interviewees 6 months earlier, whereas NB had spoken to them via telephone only. Participants knew that EE had developed the app as part of her PhD research and that NB was covering maternity leave.

Interviews were conducted in participants’ homes or a nearby NHS service, ranged from 7 to 53 min (mean 20 min), and were audio-recorded. Immediately afterward, the interviewer made notes in a reflective journal (eg possible themes, topic guide additions, and contextual details). In 3 cases, a nonparticipant was also present in the interview, in accordance with the study’s lone-worker policy. The researcher telephoned 1 participant shortly after the interview to clarify a point they had made. However, because of time limitations, transcriptions were not returned to participants for comment and participants were not asked to give feedback on the findings. Data saturation was not reached, despite all consenting, eligible individuals (feasibility study participants) taking part.

#### Framework Analysis

The research team conducted a framework analysis (Table 2) of interview transcripts and the study feasibility diary. The framework method allows a combination of deductive and inductive coding to be used [47]. This was appropriate as there were specific issues that we set out to investigate (*a priori* themes, eg, participants’ views on the item wording), while also allowing space to explore unanticipated aspects of participants’ experiences (*a posteriori* themes).

#### Reflexivity

EE is a PhD researcher investigating measurement of basic symptoms using a mobile phone app; SB (a clinical psychologist) and RD (a psychiatrist) have worked extensively with individuals with psychosis, both in a clinical and research capacity; and SB and NB are involved in several studies investigating digital health interventions (DHIs) for this population. Although the multidisciplinary nature of the research team promoted consideration of a variety of interpretations, we inevitably brought certain expectations and assumptions to the interview and analysis process, for example, an attachment to the app’s features that we had designed and hoped would be well received. Several steps were taken to minimize potential bias. Questions in the topic guide were worded in an open, neutral manner that avoided indicating researchers’ opinions. Interviewers were careful not to put pressure on participants to answer in a certain way. All ideas, insights, discussions, and decisions during data collection and analysis were documented in a reflective journal. The first author also avoided reading any closely related literature (qualitative studies of app interventions) during the analysis process to avoid biasing her interpretation.

**Table 2:** Framework analysis method with details regarding its use in this study.

Stage	Description	Procedure	Details of this stage in this study
1	Transcription	Audio-recorded interviews transcribed verbatim	EE^a^ transcribed all 16 interviews verbatim.
2	Familiarization	Read transcript and listen to audio recording	SB, RD, and NB each read 2 of the first 3 interview transcripts, and EE listened to audio-recordings and read transcripts of all 16.
3	Coding	Read transcript line by line and apply a code describing why that section is important	EE, SB, RD, and NB independently coded ≥2 of the 3 transcripts. *A priori* codes (eg, *item wording* and *clinician access*) were predefined by EE based on the topic guide (deductive coding), and inductive coding was used for other potentially relevant topics.
4	Developing a working analytical framework	Researchers compare codes and agree a set of codes for subsequent transcripts	EE, SB, RD, and NB met and discussed their coding; differences were resolved and a working analytical framework agreed. The discussion was audio-recorded for reference and noted in the reflective journal.
5	Applying the analytical framework	The working analytical framework is applied to subsequent transcripts	EE recoded the first 3 transcripts and then coded the remaining 13 transcripts and feasibility diary using the working analytical framework; NVivo was used to manage this process. The framework was updated where necessary (eg, new code needed), with changes discussed periodically with SB, RD, and NB. A final framework was agreed, and EE recoded all 16 transcripts and the feasibility diary for consistent coding across the dataset.
6	Charting data into the framework matrix	Data are summarized in a framework matrix. Illustrative quotations are included	EE charted the data into 2 framework matrices: 1 containing *a priori* codes and the second containing *a posteriori* codes. Both matrices consisted of 1 row per participant and 1 column per code, with codes grouped into provisional themes and subthemes. Draft framework matrices were discussed by EE, SB, RD, and NB and updated as necessary for the final versions.
7	Interpreting the data	Researchers keep notes on analytical insights during the analysis process. The team discusses these insights periodically and works toward an interpretation of the data	EE kept notes in a reflective journal during analysis. Analysis meetings were audio-recorded, with notes taken. EE used the reflective journal to revisit previous ideas and consolidate these with new insights during analysis. Once the final themes and subthemes had been agreed by the whole team, EE drafted a write-up of the findings. The team critiqued the draft before agreeing a final version.

^a^All initials used in this table are author initials.

## Results

### Stage 1: Basic Symptom Item Pool

The final wording of the 56 self-report basic symptom items is presented in Table 3, with corresponding SPI-A item numbers. Beta testers and longitudinal feasibility study participants generally reported that the items had face validity and the item wording was acceptable. A total of 6 changes in response to feedback from participants in this study and piloting in nonclinical samples are indicated in Table 3.

### Stage 2: ExPRESS App Development

#### Beta Testing

Demographic and clinical characteristics of patient beta testers and changes made to ExPRESS in response to their feedback are shown in Tables 4 and 5, respectively. Aside from the comments summarized in Table 5, beta testers were extremely positive about the ExPRESS app.

### Stage 3: 6-Month Feasibility Study and Qualitative Interviews

#### Participants

The demographic and clinical characteristics of participants are shown in Table 4, with basic characteristics of individual participants shown in Table 6 and retention shown in Figure 1. Participants were mostly white British, single, unemployed, and living alone, with a diagnosis of schizophrenia and an average age of 38 years. In total, two-thirds of the sample was male and almost half had accessed further or higher education. Although average PANSS positive scores suggest mild to moderate symptoms, these were nonnormally distributed with some participants reporting virtually no positive symptoms and others reporting much higher symptom levels.

#### A Priori Themes: Long-Term Acceptability of App Use

*A priori* themes and subthemes are outlined in Multimedia Appendix 3. Participants found ExPRESS acceptable: they were generally very positive about the specific aspects of the app discussed in qualitative interviews and most would be willing to use it weekly for more than 6 months as part of their day-to-day life.

**Table 3:** Basic symptoms checklist item pool, with corresponding Schizophrenia Proneness Instrument Adult Version item numbers.

Schizophrenia Proneness Instrument Adult Version item number	Basic symptom checklist item wording
A1.1	Doing new things has been more stressful than usual
A1.2	Crowds or people have been more stressful than usual
A1.3	Doing things in a hurry has been more stressful than usual
A2.2	I have felt empty and flat
A3	I seemed to care less about people than I usually do
B1	I have found it very hard to do two things at once
B2	I have found myself more easily distracted than usual
B3	My concentration has been worse than usual
B4	I have been forgetting things I’ve done less than an hour before
B5	My thoughts have been slower than usual
B6	I haven’t had the energy for thinking
C1	Even simple choices have been difficult
C2	Random thoughts have popped into my head
C3	My mind has sometimes gone blank
C4	It’s been hard to follow what people say
C5	I have found it hard to say what I mean
C6	I forgot things I was told almost immediately
D1	It’s been hard to decide what I’m feeling
D2	I have felt more emotional about everyday things
D3	My head’s been buzzing with lots of thoughts
D4	Random things seemed to have a personal meaning for a moment
D5	People have looked somehow different than usual
E1-E5	I’ve had some unusual feelings in my body
E6	It sometimes felt like part of my body had swollen up or shrunk
F1	Light has seemed very bright
F2	I have sometimes seen flashes of light
F3	Things have looked the wrong size
F4	I have noticed sounds more than usual
F5	Sounds have seemed different than usual
F6	My body has sometimes felt like it didn’t belong to me
O1^a^	Sometimes thoughts and images that are unimportant and have no special meaning keep repeating over and over in my mind and I can’t push them away
O2.1	Sometimes I have mixed up real and imaginary things
O2.2	Sometimes I have mixed up real and imaginary memories
O3^a^	Sometimes I take things literally when they are not meant that way. For example, I sometimes misunderstand sayings or metaphors
O4.1	Things seemed closer or further away than they actually were
O4.2	Things have seemed to change shape
O4.3	Colors have seemed different than usual
O4.4	Sometimes when I looked in the mirror I looked different
O4.5	Things that I saw sometimes seemed to move
O4.6^b^	Things have looked wonky or like there was more than one
O4.7	Judging distance or size has been hard
O4.8	Lines have looked somehow wrong
O4.9	If I stared at something and then looked away I could still see it afterwards
O4.10	I have had “tunnel vision”
O5.1	I could sometimes hear sounds that didn’t seem quite real
O5.2^a^	Sometimes I hear sounds which I heard a few minutes ago or even hours before
O5.2	Sounds have sometimes seemed to continue after I know they have stopped
O6.1	Things have smelt different from usual
O6.2	Things have tasted different from usual
O6.3	Objects have felt different from usual
O7	At times I could not take my eyes off something
O8	Everything around me has seemed somehow not real
O9.1^a^	Sometimes I make certain movements even though I had no intention to, like I have lost control of my body
O9.2^a^	I have sometimes spoken without meaning to
O10	My body has sometimes got “stuck” for a short time
O11	I have had to think about things that I usually do automatically

^a^Wording updated in response to piloting in nonclinical sample.

^b^Wording updated in response to feedback from beta tester in this study. Original wording: *Things have looked crooked or like there was more than one*.

**Table 4:** Clinical and demographic characteristics of the 2 patient samples.

Characteristics		Beta testers (n=5)	6-month feasibility study (n=18)
Age (years), mean (SD)		45.8 (21.0)	37.9 (9.9)
Gender (male), n (%)		2 (40)	12 (67)
Positive and Negative Syndrome Scale positive, mean (SD)		—^a^	15.4 (5.4)
**Diagnosis, n (%)**			
	Schizophrenia	3 (60)	14 (78)
	Schizoaffective	2 (40)	4 (22)
**Education, n (%)**			
	Secondary	2 (40)	10 (56)
	Further	1 (20)	5 (28)
	Higher	2 (40)	3 (17)
**Employment, n (%)**			
	Employed	1 (20)	2 (11)
	Voluntary work	0 (0)	1 (6)
	Retired	2 (40)	1 (6)
	Unemployed	2 (40)	14 (78)
**Ethnicity, n (%)**			
	Asian or Asian British	0 (0)	1 (6)
	Black or black British	1 (20)	2 (11)
	White British	4 (80)	15 (83)
**Marital status, n (%)**			
	Single	5 (100)	14 (78)
	Married	0 (0)	2 (11)
	Separated	0 (0)	2 (11)
**Living arrangement, n (%)**			
	Alone	2 (40)	12 (67)
	With family	2 (40)	4 (22)
	Supported accommodation	1 (20)	2 (13)

^a^Beta testers were not assessed using the Positive and Negative Syndrome Scale.

**Table 5:** Changes made to the app or training protocol in response to feedback from patient beta testers.

Comment or suggestion [participant number]		Change made to the app	Added to the app training protocol
**Item wording**			
	PANSS^a^ follow-up questions are confusing; it seems like items are repeated [P1]	Change not possible; PANSS items validated	Explanation of branching follow-up questions
	Items phrased in own words may be invasive [P1]	—^b^	Check participant is happy with item wording before use in the study
	Alter *crooked* to *wonky*, basic symptom item O4.6 [P3]	Altered the wording of the item as suggested	—
	Did not realize that PANSS follow-up questions are contingent on previous answers [P5]	—	Explanation of branching items; follow-up items are contingent on response to initial item
**Item content**			
	*I had to generalize in some cases. Specifics occurred to me later* [P2]	—	Reassurance not to worry if cannot remember everything
	Items did not seem very closely connected [P2]	—	Description of item types and how they are connected
	Participant would not want to answer items about certain symptoms once unwell [P5]	Added to instructions: If you don’t feel comfortable answering, please feel free to ignore the alert	If you do not feel comfortable answering, please feel free to ignore the alert
	When items are not applicable, might wonder why you asked about them [P5]	Added to instructions: Explanation that some are standard items; they might not apply at the moment	Explanation that some are standard items; they might not apply at the moment
**Sliding bar**			
	One PANSS item is scored in the opposite direction [P1]	—	Explanation that 1 item is scored in the opposite direction
	1-7 scale felt odd; a 1-10 scale might be better [P2]	Change not possible; PANSS items validated	—
	Tended to answer to the extreme (1 or 7) [P2]	—	Suggestion to leave room for improvement or deterioration when answering items
**4-point scale**			
	Change anchor wording so that the *number of times a week* is first [P1]	Anchor wording changed as suggested	—
	Previous selection sometimes stays for next item [P3]	This bug was fixed	—
**Alerts**			
	Afternoon best as gets up late because of medication [P3]	Moved the alert time to afternoon (1.30 pm)	—
	Reminder when the 24 hours is nearly finished [P3]	Added extra reminder after 23 hours	—
	During work lunch break would be best [P5]	Moved the alert time to 1.30 pm	—
	Would like to be able to set own alert time [P5]	Not changed; consider for future app version	—
	Would like to be able to set the snooze duration [P5]	Not changed; consider for future app version	—
**Worries**			
	Worried that the items might be negative or might tell him to do something negative [P3]	—	Reassurance that the items are the same each time and will never tell you to do anything
	Worry that other people might get hold of the answers [P4]	—	Reassurance that only the research team can see the uploaded answers
	Worry that someone picking up phone might see psychosis mentioned in the app [P5]	—	Explanation that answers are not visible after upload; guidance on putting a lock on the phone
**General**			
	Make more accessible to standard mobile phones [P2]	—	Participants can borrow a study smartphone
	*Smartphones take some getting used to* [P2]	—	Participants without smartphone experience will need more training
	Prefer menu button to be in the top left corner [P3]	No change; not an issue for most participants	—
	A back button would be helpful [P3]	Not changed; consider for future app version	—

^a^ Positive and Negative Syndrome Scale.

^b^Cells in this table are empty in cases where the column is *not applicable*, ie, no changes were made to the app or training protocol.

**Table 6:** Basic characteristics of individual participants.

Participant	Age (years)	Gender	Baseline Positive and Negative Syndrome Scale positive subscale score^a^
P204	51	Female	20
P205	28	Female	15
P206	36	Male	7
P207	35	Male	26
P208	42	Female	11
P209	51	Male	18
P211	28	Male	16
P214	40	Female	22
P215	37	Male	18
P223	37	Male	7
P224	22	Male	21
P225	48	Male	16
P227	41	Male	19
P230	57	Male	16
P231	22	Female	9
P235	39	Male	12
P236	41	Male	16
P239	28	Female	8

^a^Possible range: 7 to 49.

**Figure 3:**
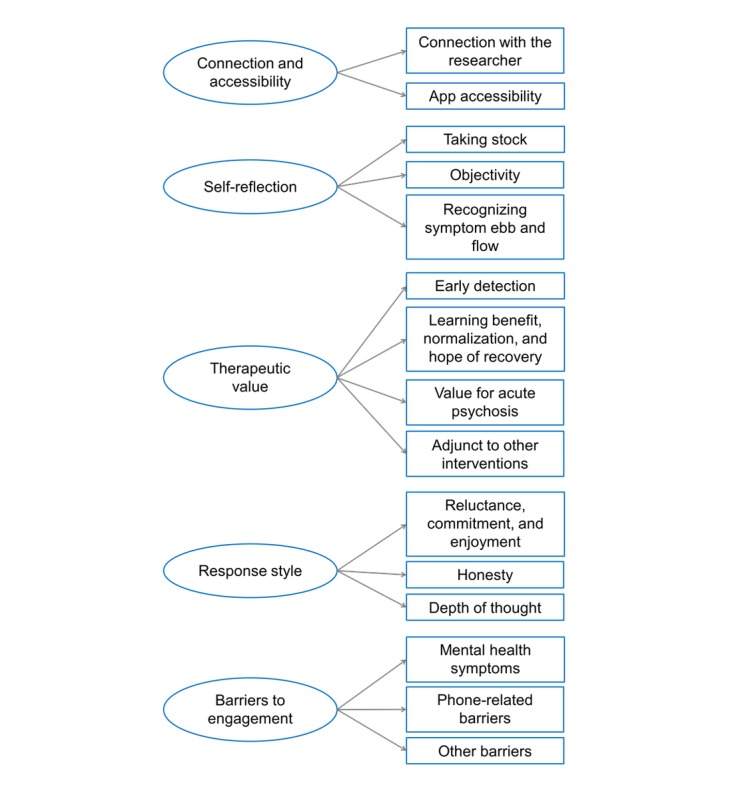
Summary of a posteriori themes and subthemes.

#### A Posteriori Themes: Benefits of and Barriers to Long-Term App Use

##### Overview of A Posteriori Themes

A total of 5 *a posteriori* themes were derived from inductive coding of interview transcripts: connection and accessibility, self-reflection, therapeutic value, response style, and barriers to app engagement. Overall, these themes relate to the perceived benefits of, and possible barriers to, app use in this population. Participants were not directly asked about these themes in the topic guide but disclosed them spontaneously when discussing their experiences of using the app during the previous 6 months. *A posteriori* themes and subthemes are summarized in Figure 3.

##### Connection and Accessibility

###### Overview

This theme brings together participants’ comments that relate to the importance of their connection with the researcher and the extent to which they found the app easy to access. Participants compared their interactions with both the researcher and the app to their interactions with mental health staff.

###### Connection With the Researcher

The connection with the researcher and a sense of belonging to the study were very important for some participants who found reminder texts and regular telephone calls positive and encouraging:

You’ve really done a lot because you are taking part every day [laughs] just reminding me every day, is part of it. It’s as if we are together!

P206

This participant completed the app assessment every week during the 6-month study period; therefore, it might be that participants’ degree of engagement with the app is dependent on the rapport with the person facilitating delivery, in this case the researcher. One individual felt it was important to know who was receiving her app answers, not just from a confidentiality point of view but also so she knew who she was talking to:

So sometimes, you’d be constantly thinking “well who are you telling all this to and what’s the point?”

P239

Filling in the app was not just a self-reflective exercise for this participant but one involving communication with a specific person.

For some participants, it took time to build a rapport with the researcher, with 2 commenting that they found the initial meeting somewhat stressful, but participating got easier once they built a rapport. One individual who was generally anxious about telephone calls was happy to receive calls during the study. The researcher noted in the reflective journal (reflective journal, data analysis phase):

Phone calls were acceptable. It probably helps that I had met all participants in person and discussed symptoms in detail face-to-face before the phone assessments. Rapport was already established and they had already shared significant info with me without me freaking out or causing them any problems

Several participants commented that it was reassuring to know that somebody was looking at their app responses:

It was just nice to know that there would be somebody looking over you...so that was quite comforting in a way. [P208]

Some seemed to view the study as almost an extension of clinical care:

I also think it depends on the individual person, how they effectively view the phone calls cos, if you have a care coordinator and your care coordinator checks up on you it’s just like that so... I don’t see it kind of different.[P205].

Conversely, 1 participant emphasized that her care coordinator did more than simply assess mental state:

She helps me with other things...so it’s nice that you’ve got someone there for you.

P239

###### App Accessibility

The majority of participants (83%; 15/18) owned a smartphone and a few mentioned in the interview that they used apps for other things, which made using an app to communicate about their mental health *a natural thing* [P205]. For example, 1 participant reported using apps such as Facebook and WhatsApp so included the app as part of his social network:

But it benefitted more than Facebook cos it’s really talking about my health.

P206

Some individuals seemed to enjoy the app because they experienced it as similar to a person:

It’s almost like somebody’s speaking to you...asking me questions like “how’s your day been?” or “how’s your week been?”

P205

It becomes like a...little friend.

P208

However, others liked it because they did not have to talk to a person, reporting:

You feel... you can open up more. I know it sounds weird.

P214

Strikingly, those who liked the app because they did not have to talk to a person had high symptoms during follow-up, whereas those who described the app as conversation-like all had low symptoms. It is possible that certain features of an app make it nonthreatening to someone who is wary of disclosing high symptoms; for example, an app might be experienced as more neutral and less judgmental than a person as it does not give any verbal or nonverbal feedback.

Participants reported other ways in which they found the app more accessible than mental health staff. One individual noted that the app asked questions more frequently and regularly than he typically saw his care coordinator, which he found useful:

They [the questions] come every week, she [care coordinator] come like four weeks, three weeks, two weeks, so, but this is reminding me of what I’m supposed to do, all the time.

P206

Another tended not to seek help from his care coordinator when he was having a bad week because he was *a bit stoic* [P236] and did not want to bother her but he still answered the app at these points, which enabled the care coordinator to offer additional help when necessary. Similarly, it was noted in the study feasibility diary that 1 participant filled in the app once during relapse at a point that he was not willing to do a telephone interview, suggesting that, for some, an app could be more accessible in cases where a patient is unwell and unwilling to talk.

##### Self-Reflection

###### Overview

Participants spontaneously reported a number of ways in which the app helped them to reflect on their own mental health. Not only did completing the app provide a time to take stock of things but it also enabled them to reflect on their experiences more objectively and to notice patterns in their experiences over time.

###### Taking Stock

The process of answering questions on the app prompted participants to take time to think about their mental health each week (*It makes you realize, you know, take sort of stock of where you’re at* [P214]), which they believed they would not have done otherwise (*Like I had to say to myself “think about your week” cos sometimes I don’t think about the week* [P239]). By eliciting regular self-reflection, the app might serve a similar function to talking to a confiding person, as sharing experiences often provides an opportunity to reflect and process them:

My mum asks me like how’s my day been or I just automatically tell her so by the time I’m coming to do the questions it’s like well I’ve already spoke to mum about this and that so it’s just a reminder of that.

P205

###### Objectivity

The process of answering the questions gave participants the opportunity to translate their subjective experiences into an objective format:

I found that the more I used it, the more I started looking objectively at my life

P205

This provided some individuals with a sense of the realness of their experiences, especially with the help of the graphs:

When you look at the analysis of the data collected...you can objectively see.

P205

Participants theorized that having access to objective data representing their symptoms, particularly in the graph form, might enable a shared understanding of their experiences, both with the care team (*Maybe help themselves to understand a little bit better* [P227]) and potentially with the general public (*If it can help people in the future understand a little bit better about what we go through* [P227]). Developing a shared understanding with the care team might facilitate help seeking. One participant who had sought help for an emerging relapse in the past but had not been taken seriously suggested:

If you were to answer the questions and go to the doctor and say “look, these are my results, you can see clearly there’s a change, and these are my experiences,” that would be substantial evidence for the doctor to then sit up and take note.

P205

###### Recognizing Symptom Ebb and Flow

Taking time to reflect every week and to articulate their experiences in an objective format helped participants notice the level of their symptoms and to recognize ways in which this varied over time, providing a long-term perspective on the ebb and flow of symptoms:

They come and pass, they come and pass away

P205

Several participants acknowledged the potential value of the app in spotting fluctuations in their symptoms:

If it’s something could spot my mood going up and down and things it might be useful.

P207

It would gauge how well you were.

P230

##### Therapeutic Value

###### Overview

Although a few people could not think of anything that they found helpful about the app when asked directly and 1 participant stated that he did not find the app particularly useful as he is *already aware of everything* [P207], the majority of participants reported finding the app beneficial.

###### Early Detection and Staying Well

If the app was used in a clinical setting, the intention would be to help patients and clinicians spot predefined early signs and intervene to prevent a full relapse from occurring. Several participants spontaneously identified this as a potentially valuable outcome of using the app (*It can be like a toolkit for you...A toolkit for like er staying well really...A wellness reaction plan you know. A “wrap”* [P230]), with 1 participant suggesting that using the app would help her and others recognize changes in their symptoms in response to stressors:

It could help people identify, cos like for me, now that I’ve used an app, I’m thinking when I know my stressors are coming I’d like to have the app there available so that I can do it and then see “oh is it like my thoughts are changing.”

P205

Recognizing patterns in their symptoms allowed some participants to take action to help themselves stay well during the study. For example, 1 participant reported that using the app meant that she recognized that she felt unwell the week before her monthly depot was due:

Cos it gets you into a pattern of realizing when you’re a bit down, when you’re a bit, you know, dodgy type thing.

P208

The realization that she was feeling down because her depot was due reassured her (*I thought that the levels in my depot was probably going down a bit and I was ready for another depot. That’s what that helped me to realize. And that helped to keep me calm by using the phone app* [P208]) and meant that she was able to use *PRN* (*pro re nata)* medication (medication that can be taken when required) at this time. Thus, using the app could help patients to take more responsibility in managing their mental health in conjunction with their care team.

###### Learning Benefit, Normalization, and Hope of Recovery

For many, the opportunity for self-reflection provided by the app was a valuable experience that increased their understanding of psychosis:

I think it probably made me understand my illness a little bit better. The voices and all.

P211

It’s been eye opening.

P208

Although, for some, this brought the more uncomfortable realization that:

I’m not as well as I thought I was.

P227

Although not formally assessed in the study, 1 participant appeared to have a sealing-over recovery style, a form of avoidance coping common in people recovering from psychosis [55]. This individual did not wish to continue using the app for longer than 6 months as he did not like being reminded of his symptoms:

I just don’t feel like looking back on myself in a way if you see what I mean.

P223

In contrast, another participant initially worried that thinking about her experiences every week would remind her that she was ill but actually, it had the opposite effect (normalization):

I thought oh it’d make you feel like oh you’re ill, you know. It’s just a constant reminder: you answer the questions and the results show that you’re ill. But, if anything, it showed that you have this illness but you’re normal, you have normal feelings and emotions and anybody can have them at any time.

P205

There were several ways in which this participant found the app normalizing: (1) it helped her to discuss symptoms in a normalizing way (*Cos when I was talking to mum about some of the questions it was like – yeah, she feels that, at times. Ok she doesn’t hear voices but she feels down and things like that and just that normalization of talking to somebody about the study and then explaining to them how it was*. [P205]); (2) the fact that there was an app for tracking symptoms was normalizing in itself because of the availability of apps for lots of everyday things; and (3) taking part in the study was normalizing as there must be other people with similar experiences (*It was like a normalization so, cos I knew I was taking part and many others but I don’t know to what degree their psychosis is but like it’s just a normalization of like well we have this psychosis.* [P205]).

This participant found that the normalizing effect of the app facilitated a sense of acceptance of her experiences, enabling integration rather than sealing over (*It was just an acceptance it’s part of me and it’s normal life. Like I didn’t differentiate it from “oh this is a part of me that I don’t know about and I don’t like.” It was just a part of me that I could just carry on living with.* [P205]), and gave her hope that she could recover (*Afterwards I’d just be like “I can get better. I can get better!” there was just, there was this hope that I had when I was using it.* [P205]). She speculated that, had she been filling in the app for a few years, seeing the pattern would have helped her to see the extent of her recovery to date:

My answers to the questions three years ago... would have been completely negative and so to see the change in that, then I would have been like “Yeah actually...I can see I am getting better.”

P205

###### Value in Acute Psychosis

Two participants commented that the app would be useful during the acute phase of psychosis:

I think it’s good for maybe someone that’s just gone into hospital.

P239

One thought that using the app during an initial episode of psychosis might help people to be more recovery-focused (*I think like if you got most people with psychosis to do it when they’re like at their worst illness... I think it would, it’d have a bearing on them being more positive about the illness and being more positive about how they could get through it, with it rather than against it*. [P205]) and that it might help mitigate memory loss associated with the acute phase:

Like for me when I was talking about my illness, the more I got ill, the more I couldn’t remember. And I, I think that’s to do with psychosis sometimes, your memory’s not so good.

P205

###### Value in Conjunction With Other Interventions

All participants in the study were prescribed a maintenance antipsychotic during the follow-up period, but 1 participant in particular [P206] found the app complemented this well in that it increased his medication adherence by providing a sense of accountability. As this participant felt that he should be honest on the app and he did not want the researcher or his care coordinator to see that he had not taken his medication, this motivated him to continue taking it:

With this app, it’s, even when I want to stop my medication, you know, I won’t tell lie that I didn’t take my medication, so it will now tell me “ah, you need to take your medication because tomorrow when you are doing this... you have to be sincere... so you should take your medication!” [P206].

Participants also noted the value of the app in conjunction with other interventions, for example, cognitive behavioral therapy (CBT) or occasional use of PRN medication.

##### Response Style

###### Overview

This theme relates to participants’ comments regarding the way they completed the app: whether they did so reluctantly or willingly, how much thought they put into their responses to the app’s questions, and whether they were honest in these.

###### Reluctance, Commitment, and Enjoyment

Participants reported that the app was sometimes annoying but sometimes helpful. Although a few people seemed to genuinely enjoy the process of completing app entries (*I did enjoy answering the questions... I didn’t see it as a chore*. [P230]), most others viewed it as a chore (*Not really anything to enjoy actually doing the app... It’s not like you’re playing a computer game or you know*. [P236]), but one which hopefully had some benefit (*The questions do help you a bit.* [P214]). Several participants expressed some reluctance at using the app at times but usually did it anyway because of finding it easier than talking about how they felt [P224] or because they were committed to taking part in the study (*I thought no I’ve agreed to do it so I’ll carry on doing it.* [P227]). Moreover, 2 participants described a process of motivating self-talk to persuade themselves to complete the app:

I just kept saying to myself “you’ve nearly finished, you’ve nearly finished, it’s not forever.”

P214

I had to say to myself “think about it...just answer the question” you know.

P239

One participant who had enjoyed the app was extremely committed to it, even describing 1 week that he returned early from staying overnight in another city specifically to do the app:

I didn’t go away with the phone so, on Thursday when I woke up in the morning I just told my friend... “I have to go back to Manchester cos I’m supposed to do this thing yesterday”... so I rushed down.

P206

###### Honesty

A number of participants commented that they answered the app honestly, despite, in some cases, feeling some paranoia about it or finding it tricky to force themselves to think about their week:

I just had to say to myself “tell the truth, think”... I had to say to myself “think about your week.”

P239

One participant reported during a telephone call with the researcher (noted in the study feasibility diary) that he had begun to answer the app more honestly: although his app responses appeared to indicate that he was getting unwell, he was in fact just being more honest.

###### Second Guessing Answers Versus Answering Without Deep Thought

Some participants appeared to put a lot of thought into answering the questions, for example, by second-guessing their own answers:

Even as I was doing it I was thinking well was that right, maybe I should have put something else, you know, different number.

P236

Conversely, others reported that they answered without deep thought:

I didn’t really think about it [laughs slightly] you know.

P235

One participant commented that he did not record small fluctuations in his mental health on the app:

I don’t allow it to stay long, so that’s why I don’t put it there.

P206

##### Barriers to App Engagement

###### Overview

Participants noted a number of barriers, which prevented them from engaging with the app more fully. Mental health symptoms and phone-related barriers interfered with app use in some cases, though these rarely prevented app engagement entirely.

###### Mental Health Symptoms Interfered

Positive symptoms interfered with app engagement in several cases, but the extent of this interference varied. One participant had very high levels of positive symptoms that he did not believe were symptoms of an illness; he did not want to continue with the app because he believed it was irrelevant to him, making him feel like an *experimental rabbit* [P215]. Other participants with positive symptoms were happy to carry on with the study but they occasionally delayed answering questions or missed them for a week and sometimes they took longer to answer:

Sometimes it takes a lot longer cos I’ve got the voices going on at me so it, it takes longer cos I have to argue with them, and I have to really concentrate on what’s on the phone...and then it’s absolutely knackering. I know it sounds weird cos all I’m doing is answering questions.

P214

Overall, 2 participants relapsed during the study. One of these individuals stopped using the app the week before he met study criteria for relapse, having used it virtually every week beforehand. Thus, for some, stopping the app might be a sign of imminent relapse, an observation that is consistent with findings of a previous study in which early signs of relapse were monitored using postal questionnaires [23]. The second relapsing participant stopped using the app the week after meeting relapse criteria, meaning that the app assessments in the previous few weeks could be used to predict relapse. Encouragingly, both participants were happy to continue with the app once the relapse had resolved:

It was only that time when er I was really unwell ...I was just so unwell that I couldn’t do it but then after that I went back into it. [P230].

Mood symptoms affected a few participants’ ability to engage with the app (*when I was really low... I just didn’t communicate with anyone anyway* [P227]). Although 1 commented that *even when things were not so good for me, it’s still easy to answer it* [P239], she pointed out that mental health staff can take into account how you are feeling when asking questions in a way that the app cannot:

When you’ve got people coming to you...they can perk you up or get through it and eventually answer the questions.

P239

She also suggested that her mental state at the time of answering the app might affect how she answered; for example, if she was feeling happy when answering them, she might forget that she was feeling down at other points in the week.

###### Phone-Related Barriers

A total of 13 people used a study phone during follow-up. Some found the study phone a barrier, stating that they did not tend to look at it regularly, did not take it out with them, sometimes struggled to use the unfamiliar handset, were worried about it being broken or stolen, and that they might forget to charge it. This issue was mitigated to some extent by the researcher sending a weekly reminder text message to participants’ own phones.

Lack of smartphone experience was a barrier in some cases, with 1 participant accidentally deleting the app from the phone and 2 others commenting that their lack of smartphone experience prevented them from accessing the app's extra features. Unlike a *digital native*, who would probably have explored the app’s features of their own accord, they only used its basic functions. One participant with little smartphone experience described a general anxiety about using phones, including for telephoning and text messaging; another commented:

Sometimes I get tired of looking at a phone.

P239

###### Other Barriers

Some participants expressed skepticism regarding certain aspects of the study such as whether the research would find anything useful (*You don’t gonna find patterns. I’m pretty sure*. [P215]), the validity of translating experiences into numbers (*You get a more of an insight I would say with erm [pause] people's opinions and thoughts and that rather than turning them into numbers and statistics.* [P236]), and how the researcher would work out what the answers on the app meant (*I just think about the other person on the other side having to sit there and read the questions. How do they work it out?* [P239]). If not addressed, such skepticism might hinder app engagement as beliefs and attitudes predict intentions and behavior [56].

Lack of literacy was a significant barrier for 1 participant and prevented him from continuing with the study:

Basically I’m not very good at reading and spelling...that’s the reason really...I didn’t really want to say last time I seen you...I felt a bit embarrassed...

P225

A number of more minor barriers were recorded in the study feasibility diary. Participants missed 1 or 2 weeks of app use because of being physically unwell, lending their phone to someone else, being busy, oversleeping, being on vacation in the United Kingdom, or being abroad. The study policy was for participants not to complete the questions when they were abroad (because of lack of local support in the destination country). However, allowing participants to do so might increase their access to help at such times as life events, even positive ones, are associated with relapse [57,58].

## Discussion

### Overview

This study describes the development and testing of ExPRESS, the first mobile phone app to monitor basic symptoms and conventional early signs of psychosis relapse. It also outlines the development of the BSC, a pool of self-report items that can be used to monitor basic symptoms as putative relapse predictors. It has the longest duration of any study testing a symptom monitoring app in a sample of individuals with established psychosis and is the first study to qualitatively examine psychosis patients’ experiences of using an app over a 6-month period. Framework analysis of interviews with patients enabled us to examine both *a priori* (acceptability) and *a posteriori* themes (wider app experiences).

### Principal Findings

*A priori* themes related to the actual acceptability of the ExPRESS app and its use in day-to-day life. Participants found the app acceptable in terms of the way it looked, length of assessments, item content, item wording, and response format. As assessments were only weekly and the 24-hour response window meant they could answer when convenient, most (including those with jobs) reported that the app fitted well with their routine and they would be happy to use it for longer than 6 months as part of their day-to-day life. This contrasts with feedback from participants using Clintouch [36], an app with several symptom assessments per day, who were unwilling to use it for longer than 2 weeks and thought it would not fit well with employment. The authors of the latter study suggest that symptom monitoring for relapse prevention might require less frequent assessments than Clintouch [36], which is consistent with recommendations of minimum fortnightly monitoring during early signs interventions [59]. This study shows that with less frequent monitoring, people are willing to use a symptom monitoring app for significantly longer.

In contrast with clinicians’ hypothetical views [39,60,61], most participants were happy with a clinician having automatic access to their responses. The absence of difficulties while using ExPRESS, which automatically uploads data to the researcher, might have mitigated concerns about automatic uploads in our sample. Indeed, a recent systematic review found that actual acceptability of various aspects of DHIs tended to be higher than hypothetical acceptability [46]. Similarly, after using a mood monitoring app for 3 months as part of the Automated Monitoring of Symptoms Severity study, participants with bipolar disorder were generally willing for clinicians to access the data [62]. However, although clinician access was viewed by participants as potentially valuable, the practical and legal implications of such access require further consideration if apps are to include this feature.

Participants in this study did not report significant worries about the app, except for 2 reporting some paranoia about it and 2 who worried about hospitalization, neither of which prevented engagement. The fact that these worries did not hinder app engagement might be because the research study and the app itself came from a trusted source, namely, a university. Previous qualitative studies with participants with early psychosis [41] have reported that endorsement by a trusted source, such as a university or health service, is likely to allay participant concerns about privacy and security issues.

Virtually no one used the app’s extra features such as the daily diary, graphs, or helpful numbers because they did not remember being told about them. Given that participants would have used the graphs had they known about them, more emphasis should be placed on these features during the app training session to ensure that participants are aware of them.

The *a posteriori* themes encapsulate participants’ wider experiences of using the app, including participants’ connection with the researcher, accessibility of the app, self-reflection, the therapeutic value of the app, participants’ response styles, and barriers to app engagement. A number of observations and inferences can be made from the first theme, *connection and accessibility*. The person who the patient sees as administering the app appears to have a key role; taking time to build up a rapport in face-to-face sessions might increase app engagement. Regular telephone calls and text messages from the researcher were important to participants, consistent with findings that the acceptability of DHIs is higher when patients have access to remote support [46,63-65] and that using technology can help patients and clinicians maintain a connection between appointments [37,66]. Reviews indicate that telephone support is acceptable to those with severe mental illness [67,68]. In this study, even a participant who was generally anxious about telephone calls found them acceptable as he had met the researcher previously. This is an important finding for researchers and clinicians seeking to assess symptoms remotely via telephone. Although it is unlikely that patients will be willing to discuss their symptoms over the telephone with a stranger, 1 or 2 meetings in which symptoms are discussed face-to-face might be sufficient for them to feel comfortable discussing them over the telephone.

Although participants found it reassuring to know that someone could see their weekly symptom reports, and some viewed this as an extension of clinical care, others emphasized that clinicians do more than simply monitor symptoms. Thus, as in previous studies with both patients and staff, participants in this study suggested that self-monitoring apps should enhance rather than replace face-to-face appointments [36,60]. Nevertheless, as participants in a recent study hypothesized [41], some found an app more accessible than mental health staff, reporting that they are more open about their symptoms on an app, they can complete it at points when they are unwilling to speak to someone, it is more frequent than their usual contact with a clinician, and it removes the feeling of inconveniencing someone to report symptom increases. Furthermore, several participants with low symptoms described the app in positive, anthropomorphic terms (eg, as a friend), supporting previous findings that individuals can develop strong connections with mobile phones [69].

Some of these qualities (eg, openness, acceptance, friendliness) overlap with descriptions of therapeutic alliance with respect to DHIs [70,71]. Those with psychosis [71] or depression and anxiety [70] have reported a positive therapeutic alliance with DHIs in the absence of a therapist. In the latter case, higher levels of alliance were associated with greater engagement in self-monitoring tasks [70], implying that therapeutic alliance might be an important consideration when evaluating symptom-monitoring apps such as ExPRESS. However, it is often difficult to disentangle the therapeutic alliance attributable to the DHI and to the person administering it [70]. Future studies using ExPRESS could examine therapeutic alliance with the app and the researcher using self-report measures such as the Mobile Agnew Relationship measure [71] and the Agnew Relationship measure [72], respectively.

The second theme, *self-reflection*, suggests that using a symptom monitoring app prompts patients to reflect regularly on their experiences and to express them in an objective way. Doing so enables them to recognize the ebb and flow of their symptoms and to share their experiences with others. This can have surprisingly therapeutic results, at least for some people, as summarized in the third theme, *therapeutic value*. Participants recognized the potential value of an app such as ExPRESS for early detection of deterioration, with many reporting that using such an app increased their understanding of their psychosis. This facilitated self-management in some cases, which can be empowering for patients [28,73,74]. It also appeared to have a normalizing effect for some, which seemed to aid an integrative recovery style.

Conversely, patients with a sealing-over recovery style might not be willing to use a symptom monitoring app as it provides regular reminders of past or current symptoms. Previous studies have framed participants’ discomfort with being regularly reminded of their symptoms as a side effect of the *biographical disruption* experienced by individuals coming to terms with a diagnosis of a chronic disorder [36]. These 2 explanations tap into the idea that regular symptom monitoring reminds patients of their symptomatic status, which can threaten their already fragile view of themselves. They are consistent with findings that a sealing over recovery style predicts low engagement with mental health services more generally [75] and with findings from patients with bipolar disorder [63,76].

Particularly for early psychosis patients, an app is more like their usual communication with others making it potentially destigmatizing compared with usual care [41]. Participants emphasized the potential value of the app for those in the acute phase of psychosis. However, such individuals might require additional support with app use as acute patients do not engage with symptom monitoring apps as well as remitted patients [26]. Nevertheless, with support, using an app during the early stages of a first episode of psychosis might prompt a more recovery-focused approach.

Participants also underlined the potential value of using a symptom monitoring app such as ExPRESS in conjunction with other interventions such as maintenance antipsychotics and CBT. Indeed, self-monitoring is by no means a new concept and has long been used in the context of CBT as a means of fostering *collaborative empiricism*, with its therapeutic effects reported across a wide variety of psychological disorders [77]. Although often used clinically, there was a decline in self-monitoring research since the 1980s [78]. Nevertheless, the advent of mHealth has seen this revived to some extent [79]. For example, a recent randomized controlled trial comparing app-based symptom monitoring (Clintouch app) with usual care found a significant reduction in positive symptoms in the early psychosis subsample, though not in a sample with chronic psychosis [80]. Similarly, studies using mHealth to prompt medication adherence have had promising results [81,82].

The fourth theme, *response style*, indicates that patients are generally willing to use a symptom monitoring app, if they can see the benefit, despite it being a chore. Most participants in our study reported answering honestly, despite concerns raised by patients and staff in previous studies that people might underplay or overplay their symptoms on the app to elicit or reduce care [36,60]. Nevertheless, this study also suggests that, if a patient reports increased symptoms, it is worth considering the possibility that they have begun to answer more honestly rather than that their symptoms have actually increased.

The final theme, *barriers to engagement*, summarizes several factors, which might hinder app engagement; it is useful to identify these so that they can be addressed to increase app engagement in future studies and potentially in clinical practice. Although both psychotic symptoms and mood symptoms interfered with app use to some extent, especially in patients lacking insight, most individuals with high symptoms engaged well with the app, albeit more slowly than others. Similarly, a recent study [33] found that baseline psychotic or depressive symptoms did not predict app completion rates, although negative and agitation symptoms did in a subgroup of study completers, although Ben-Zeev et al [29] reported that app completion was not associated with cognitive functioning, negative symptoms, or persecutory ideation. These findings contrast with surveys of digital health experts, who suggested that poor cognitive functioning and severe symptoms might negatively impact engagement with DHIs [83].

Use of a study phone was reported as a barrier by some, as participants did not tend to integrate this with their daily life as they would their own phone. Although previous qualitative study participants have also expressed a preference for their own phones [27,36], a recent study in an early psychosis sample found no difference in completion rates between those using study phone and those using their own phone [33]. Other key barriers that could be addressed to increase app engagement included lack of smartphone experience, skepticism regarding the app, the study policy to not use the app while abroad, and lack of literacy. Although the latter was also reported as a potential barrier by a third of participants in a recent qualitative study, they nevertheless found mobile phones more accessible than paper-based alternatives [41].

### Strengths and Limitations

The range of clinical histories within the sample is an important strength of the study; there was a substantial proportion of participants with longstanding illness and high levels of residual symptoms and a wide age range. Precautions were taken by the researchers to avoid biasing their interpretation during the interview and analysis process.

A number of limitations should be borne in mind when considering the results of this study. The sample size was modest, and despite the range of participants, this is not a representative sample, so findings should be interpreted with caution. Participants had all consented to use an app as part of a research study so they might be an unusual subset of patients who are particularly interested in mHealth. We did, however, seek to interview all 4 people who dropped out of the study; 2 were uncontactable but the remaining 2 were interviewed. It is possible that socially desirable responding might have biased reported views as half the participants were interviewed by the researcher who conducted app training and all other study procedures. This said, half the interviews were conducted by a new researcher, potentially reducing this bias. The mean interview length was relatively short for a qualitative study, but there was a wide range. Nevertheless, as illustrated by the reported participant quotations, the study was still able to gather rich, detailed information about patient experiences of using ExPRESS.

### Future Research

Having demonstrated the long-term acceptability (reported here) and feasibility (reported elsewhere) of the ExPRESS app, the next step will be to conduct a longitudinal study with sufficient power to examine the hypothesis that adding basic symptoms to conventional early signs improves relapse prediction. If such a study shows that basic symptoms do predict relapse, the app can then be tested as a clinical tool.

### Conclusions

Symptom monitoring apps have the potential to help services move toward more preventative strategies, in which symptom deteriorations are tackled early, rather than reactionary strategies, in which deteriorations are only addressed once they warrant crisis intervention. Although high caseloads often limit the frequency of face-to-face appointments, this study suggests that participants find it acceptable to use technology (phone calls, texts, and a symptom-monitoring app) to maintain contact between face-to-face meetings. Although undoubtedly many clinicians already adopt similar strategies with younger patients, this study suggests that it is possible and acceptable to do so across a wide age range, including with older patients who are less familiar with technology. As long as the potential benefits are understood, patients are generally willing and motivated to use a weekly symptom monitoring app; virtually, all participants in this study were prepared to do so for more than 6 months.

## Appendix

Multimedia Appendix 1 App evaluation form used during Stage 2 beta testing.

Multimedia Appendix 2 Topic guide used during Stage 3 qualitative interviews.

Multimedia Appendix 3 A priori themes and subthemes: long-term acceptability of Experiences of Psychosis Relapse: Early Subjective Signs.

## Abbreviations

BSC: Basic Symptoms Checklist
CBT: cognitive behavioral therapy
CDS: Calgary Depression Scale
DHI: digital health intervention
DSM-IV: Diagnostic and Statistical Manual of Mental Disorders, 4th Edition
ESS: Early Signs Scale
FoRSe: Fear of Recurrence Scale
mHealth: mobile health
NHS: National Health Service
PANSS: Positive and Negative Syndrome Scale
PRN: As needed (from Latin, pro re nata)
SPI-A: Schizophrenia Proneness Instrument, Adult Version
