# Supplementary material for: Development and Long-Term Acceptability of ExPRESS, a Mobile Phone App to Monitor Basic Symptoms and Early Signs of Psychosis Relapse
Source: JMIR Mhealth Uhealth. 2019 Mar 29;7(3):e11568. doi: 10.2196/11568 (PMC6460313; doi:10.2196/11568)
Supplement: Multimedia Appendix 1 [file mhealth_v7i3e11568_app1.docx]

Multimedia Appendix 1. App evaluation form used during Stage 2 beta testing

|  | **Yes** | | **To some extent** | | | **No** | **Comments** | |
| --- | --- | --- | --- | --- | --- | --- | --- | --- |
| ***What were your first impressions of the app?***  Did you like the way the app looked? |  | |  | | |  |  | |
| ***What did you think about the way the questions were asked in the app?***  Did the questions make sense?  (If not, which ones are confusing?) |  | |  | | |  |  | |
| Was it easy to follow the way they are laid out?  - Slider  - 4 point scale |  | |  | | |  |  | |
| Was it easy to complete them? |  | |  | | |  |  | |
| Do you think it’s important that the questions can be personalized (e.g. phrased in your own words)? |  | |  | | |  |  | |
| ***How did you find the length of the assessment?***  How long did it take to complete? | |  | |  |  | | |  |
| Do you think it would be ok to complete all the questions in one session? | |  | |  |  | | |  |
| Would you be willing to complete the questions every week? | |  | |  |  | | |  |
| Would you be willing to complete them every 2 weeks? | |  | |  |  | | |  |
| Would you be willing to complete the questions for 6 months? | |  | |  |  | | |  |
| Would you be willing to complete them for 12 months? | |  | |  |  | | |  |

|  | **Yes** | **To some extent** | **No** | **Comments** |
| --- | --- | --- | --- | --- |
| Would anything worry you about using the app? What in particular? |  |  |  |  |
| Can you think of anything you would change about the app if you could? |  |  |  |  |
| What day and time would you find most convenient to complete the questions? |  |  |  |  |
| Does the amount and method of financial reimbursement sound reasonable?  - £10 shopping vouchers for each Phase completed  - £10 phone credit per month for 6 months in Phase 3 |  |  |  |  |
| What type of phone do you have? |  |  |  |  |
| Do you have any other suggestions or feedback? |  | | | |
