# Supplementary material for: Development and Long-Term Acceptability of ExPRESS, a Mobile Phone App to Monitor Basic Symptoms and Early Signs of Psychosis Relapse
Source: JMIR Mhealth Uhealth. 2019 Mar 29;7(3):e11568. doi: 10.2196/11568 (PMC6460313; doi:10.2196/11568)
Supplement: Multimedia Appendix 2 [file mhealth_v7i3e11568_app2.docx]

Multimedia Appendix 2. Topic guide used during Stage 3 qualitative interviews

**Using the phone app**

- Could you tell me about your experience of using the phone app?
- What did you think about the way the questions were asked in the app?
- Did any of the questions stand out to you particularly?
  - Prompt: any particularly relevant/helpful?
  - Prompt: any particularly irrelevant/difficult?
- What do you think about the way the app looks?
- How did you find the length of the assessments? (How long did it take you to complete?)
- You’ve used the app for 6 months – what was it like using it for this long? How would you feel about using it for longer?
- How did filling in the app fit in with your routine?
- When did you tend to fill in the app questions?
  - Did you fill it in straight after the first beep, in response to one of the snooze beeps or at other times?
  - What did you think about the snooze feature (1/2 hour snooze; next day snooze)?
  - What do you think about having an extra reminder in the evening?
- Can you think of anything you would change about the app if you could? Could you tell me a bit about that please?
- Did anything worry you about using the app? What in particular?
- Was there anything about the app that you found particularly useful?
- Did you use any of the extra features on the app (daily diary, wallpaper, helpful numbers, graphs)? If so how did these help (or not help)?
- How would you feel about using an app like this in everyday life?
- How would you feel about your care co-ordinator seeing your answers to the questions if the app was used as part of your care from the clinical team?
- Is there anything else you’d like to tell me about using the app?

**Study participation**

- Can you tell me about how you got involved in this study?
- What were your reasons for taking part?
- Could you tell me a bit about what you enjoyed about taking part in the study?
- Was there anything that you didn’t enjoy? Can you tell me a bit about that?
- Did you feel you got enough support from the researcher during the study? Is there anything else that would have helped?
- How did you feel about getting phone calls from the researcher as well as filling in the app?
  - Did you feel that you should be reimbursed for the phone calls? (If so, how much?)
- During the study we began reimbursing participants £5 shopping vouchers for their time each time they completed a phone interview. How would this affect your participation in the phone interviews?
- Could you tell me a bit about why you decided to stop filling in the assessments? [if applicable]
  - *Was it for a reason related to the study?*
    - *Was there something about the app itself that was a barrier?*
    - *Was there a study procedure that was a barrier?*
  - *Was it for an external reason (unrelated to the study)?*
    - *Did symptoms interfere with using the app? [e.g. beliefs about technology]*
    - *Was it something to do with the phase of illness? [e.g. relapsed]*
- Is there anything that would have helped you to continue? [if applicable]
- Is there anything else you’d like to tell me about taking part in the study?
