# Supplementary material for: Development and Long-Term Acceptability of ExPRESS, a Mobile Phone App to Monitor Basic Symptoms and Early Signs of Psychosis Relapse
Source: JMIR Mhealth Uhealth. 2019 Mar 29;7(3):e11568. doi: 10.2196/11568 (PMC6460313; doi:10.2196/11568)
Supplement: Multimedia Appendix 3 [file mhealth_v7i3e11568_app3.docx]

Multimedia Appendix 3. *A priori* themes and subthemes: long-term acceptability of Experiences of Psychosis Relapse: Early Subjective Signs.

| Description | | Quotes [participant number] | Changes to app or training, or clinical implications |
| --- | --- | --- | --- |
| **Items: look and feel of the app** | | | |
|  | All participants liked the way the app looked; the colors were good | *I thought it was laid out nice* [P224]; *Good colors, good for the eye* [P215] | —^a^ |
| **Items: content** | | | |
|  | Participants found all or most of the items relevant; only 2 participants were ambivalent about item content | *It’s helpful...it’s relevant to what is happening to me* [P206] | — |
|  | The option to personalize the items was important | *Psychosis can be different for everybody* [P205] | — |
|  | The assessment was sometimes repetitive; some participants felt this was to trick them | *Pissed me off er that you know the repeat questions* [P215]  *Maybe they're checkin-, double checking me* [P239] | Fix technical glitches causing repetition. Remove similar items. Reassure participants: repetition is not to trick them. |
| **Items: response format** | | | |
|  | Most participants found the response format easy to use | *It was so easy... it was just a matter of pressing buttons, sliding it across* [P227] | — |
|  | 1 person found the reverse scored item (*I have felt upbeat about the future*) confusing | *Cos... it was like inverted to what the normal questions were* [P236] | Item already validated in the ClinTouch study so is not easily changed. Highlight this item during training and provide extra practice |
|  | 1 participant did not like turning his experiences into numbers | *You get a more of an insight I would say with... people’s opinions... rather than turning them into numbers and statistics* [P236] | — |
|  | No other participants had reservations about using numbers to represent their experiences. For one, this was a highlight of app use | *It was handy just writing on the number... my support worker... they ask me a lot of sometimes personal questions... and I don’t really, like, answer. I don’t like talking about my feelings... I prefer it when I just scale it on a number* [P224] | — |
| **Items: wording** | | | |
|  | All participants found the item wording acceptable and easy to understand | *I think they were asked in the right, proper right way really* [P230]  *I could understand it perfectly* [P235] | — |
|  | 1 participant commented that the Positive and Negative Syndrome Scale grandiosity question (*Compared to the average person I am*, anchor: worse, better) was confusing | In response to a question regarding difficult-to-answer questions: *Yeah probably the...do you feel worse or better* [P223] | Item already validated in the ClinTouch study so is not easily changed. Highlight this item during training and provide extra practice |
| **Items: length of assessment** | | | |
|  | All bar one found the length acceptable. Most took 5 mins; 2 took 15-20 mins, but said it was not too long | *I liked it cos it wasn’t too long* [P224] | Median time to answer questions: 5 mins (range 5-20 mins) |
|  | 1 participant felt it was too long; another suggested a progress bar | *No more than five minutes but sometimes it felt like a lot longer... sometimes I did think “oh my god this is just ongoing”* [P214] | Add a progress bar to the app indicating how much of the assessment is left |
|  | Some found they got quicker at the app as time went on | *The first time it took me a long time... second time I just knew this is same question I’ve been answering* [P206] | — |
| **App use in daily life: alerts and snooze** | | | |
|  | Participants found the alerts helpful | *I like that it notified me when I had to do it* [P224] | — |
|  | 1 participant struggled to hear the beep, so appreciated that the visual alert remained on the screen | *I didn’t always hear it at first, but then I’d look on my phone and the screen would be full of it* [P208] | Make the beep louder if possible |
|  | Only 4 participants used the snooze feature on 1 or 2 occasions each | — | — |
| **App use in daily life: clinician access** | | | |
|  | Most participants were happy with clinician access. Several described an open, honest relationship with their care coordinator | *I think that’s a really good idea* [P208]  *Well if people wanna keep tabs on me... I spose it’s useful* [P235]  *There’s nothing she don’t know anyway* [P214] | — |
|  | Participants suggested advantages of care coordinator access, including avoiding in-depth conversations about their symptoms, providing a shared understanding of experiences, and as a memory prompt | *I don't mind... my care coordinator seeing it cos in some ways it’s gonna give... representation and extra insight into how I’ve been in the past week... as well as, you know, the appointments that I have where I might forget things* [P236] | — |
|  | 1 participant was not willing for clinician access | *I think now today people are trying to like set everyone up so everyone’s monitored* [P239] | Need specific consent before giving clinicians access to the app data |
|  | 1 participant wanted to limit access to 1 individual clinician | *It would be a bit intrusive... sometimes you don’t like the ...the way you’re feeling anyway... you just don’t want everyone to know* [P223] | Consent for clinician access might need to be limited to 1 individual clinician for some |
| **App use in daily life: use in daily life** | | | |
|  | Most participants would use the app in daily life | *Yeah I’d use it. If I had the app I’d use it yeah* [P224] | — |
|  | 2 participants would not use the app in daily life: one thought it was good for research but not daily life and one would not use the app because his priority was sorting out his (delusional) situation | *I think it’s good for research but not for something for people to have constantly* [P239]  *Probably I’d not use it every day. Because... I have a big issue you know... How to sort that things with the guys which annoying and demolition my life! So... that is you know big question in my head* [P215] | App use needs to be a choice; some individuals might not want to use an app |
|  | 1 participant found it difficult to decide whether he would want to use the app outside a research context | *In some senses... it would help them give me the best care... but... it’s whether I’d get tired of answering the same repetitive questions* [P236] | — |
| **App use in daily life: duration of app use** | | | |
|  | Most participants would be happy to use the app for longer than 6 months | *I really really enjoyed it. So I say, you should don’t stop this, you should continue* [P206] | — |
|  | Several commented that the 6 months had seemed very quick | *I didn't even realize it was that long! [laughs]. It's flown by innit?* [P207] | — |
|  | 1 participant would not want to use it for longer | *I just don’t feel like looking back on myself in a way* [P223] | — |
|  | 2 expressed ambivalence about using it for longer | *Quite a large chunk out of someone’s life isn’t it really* [P230]; *Tedious* [P214] | Individuals should have a choice regarding how long they use the app for |
| **App use in daily life: fit with routine** | | | |
|  | Frequency was acceptable (maximum weekly). Most participants found the app fitted well with their daily routine because it was brief and had a 24-hour response window | *Once a week was fine but not really any more than that* [P224] | Assessments should be no more than weekly |
|  | One participant found the response window frustrating and would prefer to self-initiate responses | *They give you till Thursday to answer it but then if you’ve... missed it by ten minutes that’s it and you’re just like “oh for god’s sake”... I think if... you could answer it whenever you chose to that would be good* [P214] | Consider allowing participants to self-initiate responses |
|  | Most participants answered the app straight away or within a few hours of the beep. One participant always answered in the evening, after work | *The stresses from work will kind of influence your decisions, more, cos you’re in that situation and you’re just thinking “ah I need to do this” but when you’re at home and there’s no stress and you’re kind of relaxed* [P205] | An evening reminder might be helpful to some |
|  | For 1 participant, the app provided a routine | *At least every Wednesday I know I’m gonna do something... I’ve scheduled it, I’ve scheduled this as part of what I do every Wednesday* [P206] | Some might find the structure provided by an app helpful |
| **Other app experiences: worries about the app** | | | |
|  | Most participants did not report any significant worries about using the app. One participant suggested providing a feedback button | *Absolutely fine. I had no qualms* [P235] | Include a feedback button for individuals to indicate how they felt about filling in the app |
|  | Two participants were worried that if their symptom reports on the app increased, they might be hospitalized | *I thought things might have been held against me... like if I said like I’ve been hearing voices... I was thinking well if I put that and the wrong person sees it will I get put back in hospital?... you know cos I’m having them sensations. So I was a bit wary like that* [P227] | Reassure patients during training that no one will be admitted to hospital without a face-to-face assessment |
|  | Two participants felt paranoid about the app at some points, but this was only occasional and they were able to use the app paranoia-free at other points | *Sometimes, I don’t know why, I just feel like you know I’m giving too much away or I get paranoid about what people are going to think about my answers and erm how my answers are gonna be used* [P236] | Normalize paranoia about the app but reassure patients that it is okay to miss a week if necessary |
| **Other app experiences: extra features** | | | |
|  | One participant used the graphs and found them helpful | *I think that’s what’s needed in psychosis, somebody to objectively explain something and if they have figures... you can say “well look, this is how you’ve been feeling”... and you can then understand “yes I have been feeling like this” and “yes I can see a pattern”* [P205] | — |
|  | Most participants did not know about the graphs but would have used them had they known about them | *I have an app for [company], for my... gas and electric... gives you a graph...so you can see how much you’ve used or whatever... it’s better to have the option to look at it than for it not to be there, no option* [P236] | Emphasize the graphs more during training. Provide written instructions to remind users about app features |
|  | One participant used the daily diary. She would prefer it to upload but did find it useful for her own reference | *I think the reason that I didn’t use it towards the end was because I found out it wasn’t uploading... and I thought well there’s not really much point if nobody’s reading it... but I liked it as well. Cos if I was having like a good day I could fill it in and then look back if I was having a bad day* [P208] | Remove daily diary as not generally used |
|  | Participants liked the wallpaper in the app, although only 3 participants actually changed it | *I liked the wallpapers that you could get on there* [P208] | — |
|  | No one used the helpful numbers | — | — |

^a^ Cells in this table are empty in cases where the column is *not applicable*, ie, no changes were made to the app or training protocol and no clinical implications are noted.
